# Assessing Pediatric Growth Patterns in the Dominican Republic and Guatemala Using WHO Standards

**DOI:** 10.51894/001c.164377

**Published:** 2026-07-31

**Authors:** Paul Vigil, Logan Bunch, Zachary Kam, Nathaniel Wang, Rene Hinojosa

**Affiliations:** 1 College of Osteopathic Medicine, Michigan State University

## 40

### Background

In 2006, the World Health Organization (WHO) published the Child Growth Standards, which are now used by clinicians globally to assess appropriate pediatric development. Despite establishing an international benchmark for growth, they did not adequately account for social determinants of health or fully represent the diversity of pediatric populations. Among the several population groups not included in this analysis, the Latin American and Caribbean citizens were noted to have a prominent lack of representation.

### Objectives

The goal of this study is to collect anthropometric data for the pediatric population in the Dominican Republic (DR) and Guatemala, to analyze if the growth measurements of these children match the guidelines set by the World Health Organization.

### Methods

Anthropometric data were collected from native Dominican and Guatemalan children ranging in age from birth to five years as part of this study. Measurements were taken using the WHO guidelines to evaluate pediatric growth. Anthropometric data, including weight, length, head circumference, mid-upper arm circumference, and triceps skinfold thickness, were collected from 39 children in the DR and 30 children in Guatemala. Patient demographics along with key behavioral and social components were reported and correlated with anthropometric findings. The WHO AnthroCalc 2.0 app and Bland-Altman plots were used for data interpretation and analysis. Differences between populations were assessed with the t-test.

### Results

Most DR children had BMI-for-age within the WHO normal range (66.6%), though moderate (23.1%) and severe wasting (12.8%) were common. Measured weights for DR children were lower than WHO expectations. Bland-Altman plots show an overall bias of +1.33kg. Compared with Guatemalan children, DR children exhibit significant lower weight-for-height Z scores (t =-4.93, *p* < 0.001) but higher height-for-age Z scores (t =3.69, *p* < 0.001).

### Conclusion

Although WHO growth charts capture general trends, they do not completely reflect regional growth differences. DR children showed substantial wasting using WHO measurements, and when compared to Guatemalan children, DR children were thinner for height but taller for age. Our findings underscore the need for region-specific adjustments to reflect the specific population characteristics of each region.

